# miR-7a/b attenuates post-myocardial infarction remodeling and protects H9c2 cardiomyoblast against hypoxia-induced apoptosis involving Sp1 and PARP-1

**DOI:** 10.1038/srep29082

**Published:** 2016-07-07

**Authors:** Rui Li, Hai-hua Geng, Jie Xiao, Xiao-teng Qin, Fu Wang, Jun-hui Xing, Yan-fei Xia, Yang Mao, Jing-wen Liang, Xiao-ping Ji

**Affiliations:** 1 Department of Health Care, China-Japan Friendship Hospital, Ministry of Health, Beijing, China; 2 The Key Laboratory of Cardiovascular Remodeling and Function Research, Chinese Ministry of Education and Chinese Ministry of Public Health, Qilu Hospital of Shandong University, Jinan, Shandong, China

## Abstract

miRs (microRNAs, miRNAs) intricately regulate physiological and pathological processes. Although miR-7a/b protects against cardiomyocyte injury in ischemia/reperfusion injury, the function of miR-7a/b in myocardial infarction (MI)-induced cardiac remodeling remains unclear. Here, we sought to investigate the function of miR-7a/b in post-MI remodeling in a mouse model and to determine the underlying mechanisms involved. miR-7a/b overexpression improved cardiac function, attenuated cardiac remodeling and reduced fibrosis and apoptosis, whereas miR-7a/b silencing caused the opposite effects. Furthermore, miR-7a/b overexpression suppressed specific protein 1 (Sp1) and poly (ADP-ribose) polymerase (PARP-1) expression both *in vivo* and *in vitro*, and a luciferase reporter activity assay showed that miR-7a/b could directly bind to Sp1. Mithramycin, an inhibitor of the DNA binding activity of Sp1, effectively repressed PARP-1 and caspase-3, whereas knocking down miR-7a/b partially counteracted these beneficial effects. Additionally, an immunoprecipitation assay indicated that hypoxia triggered activation of the binding activity of Sp1 to the promoters of PARP-1 and caspase-3, which is abrogated by miR-7a/b. In summary, these findings identified miR-7a/b as protectors of cardiac remodeling and hypoxia-induced injury in H9c2 cardiomyoblasts involving Sp1 and PARP-1.

Cardiovascular diseases are the most common cause of death worldwide; an estimated 620,000 Americans have had a new coronary attack, which is defined as the first hospitalized myocardial infarction (MI), or have died from coronary heart disease^1^. During MI, maintaining efficient cardiomyocytes as best as possible is critical for the preservation of cardiac structural integrity and function because oxidative stress, along with insufficient oxygen and blood supply to the heart, leads to the irreparable loss of cardiomyocytes, and this damage is exacerbated by the toxic substances released from dead cells^2^,^3^. In addition to these immediate biological reactions, the dead cells and toxic substances trigger left ventricular remodeling, which eventually leads to functional decomposition and heart failure (HF)^4^.

As endogenous regulators of multiple target genes, miRs (microRNAs, miRNAs) are ubiquitously expressed, single-stranded non-coding RNAs with a length of 17–24 nucleotides; they regulate their targets by inhibiting their translation and/or by promoting degradation of their mRNA^5^. In recent years, miRs have been suggested as candidates in the treatment of MI-induced cardiac remodeling that may result in heart failure. miR-7 is an evolutionarily conserved mRNA in mammals, and miR-7a/b (miR-7a and miR-7b) are the two subtype of miR-7, which differs by a single nucleotide in the mice^6^. miR-7a/b is an evolutionarily conserved mRNA in mammals, and functions in growth^7^, migration and metastasis in tumor cells^8^ and fibrosis in fibroblasts^9^. Among cardiovascular diseases, miR-7 was first reported to be associated with the risk of coronary artery disease^10^ and then found up-regulated in H9c2 cardiomyoblast during I/R^11^ while no changes in the plasma of the patient suffered HF^12^. Further miR-7a/b was found to be capable of protecting cardiomyocytes against apoptosis in I/R injury by targeting poly (ADP-ribose) polymerase (PARP-1), which was over-activated and harmed the myocardium during I/R and MI^11^,^13^,^14^,^15^,^16^,^17^. However, the role of miR-7a/b in post-MI remodeling has rarely been reported.

Specific protein 1 (Sp1) is an 875-amino-acid, 100- to 110-kDa nuclear transcription factor that is widely distributed and modulates the expression of diverse genes that require one or more Sp1 binding site for activation^18^,^19^,^20^. Growing evidence has demonstrated that Sp1 plays an important regulatory role in apoptosis^20^,^21^, fibrosis^22^,^23^,^24^, inflammation^25^,^26^, and other pathologic disorders. Interestingly, PARP-1 proximal promoter activity is primarily but not entirely dependent on Sp1^19^,^27^, and Sp1 appears to be another predicted target of miR-7a/b. Nevertheless, in the present study, we evaluated the function and possible mechanism of miR-7a/b on post-MI remodeling and hypoxia-induced H9c2 cardiomyoblasts impairment. In particular, we focused on the regulation of Sp1 by miR-7a/b, with the goal of identifying potential new therapies for ischemic heart failure.

## Methods

### Animals

All experiments conformed to the Guide for the Care and Use of Laboratory Animals published by the US National Institutes of Health and Shandong University. The study protocol was approved by the Institutional Ethics Committee of Shandong University.

The sham control group underwent surgical opening of the chest without left anterior descending artery (LAD) occlusion, MI was induced by LAD occlusion, and lentiviral vector injection was performed in the 5 MI groups in male C57BL/6 mice (Vital River Laboratories, Beijing, China), as previously described^28^,^29^. Briefly, 1.5% isoflurane was used for anesthesia, and the mice then underwent LAD occlusion surgery, followed by 3 injections to deliver a total of 20 ul GFP-labeled lentiviral vectors (GenePharma, Shanghai, China) around the ligated spot. The vectors separately carried nonsense siRNA (GFP-NC), miR-7a mimics (GFP-7a), miR-7b mimics (GFP-7b), miR-7a inhibitors (GFP-anti-7a) or miR-7b inhibitors (GFP-anti-7b). Finally, we closed the chests and fed the mice regularly under standard temperature and humidity conditions in the IVC center of the Animal Care Center of the Key Laboratory of Cardiovascular Remodeling and Function Research at Shandong University. Every effort was made to minimize the suffering of the animals.

The miR-7a/b mimic sequences were: 5′-UGGAAGACUAGUGAUUUUGUUGU-3′/5′-UGGAAGACUUGUGAUUUUGUUGU-3′. The miR-7a/b inhibitor sequences were: 5′-ACAACAAAAUCACUAGUCUUCCA-3′/5′-ACAACAAAAUCACAAGUCUUCCA-3′. The scramble control miRNA sequence was 5′-UUCUCCGAACGUGUCACGUTT-3′.

### Cardiac function measurement

Transthoracic echocardiography was performed using a high-resolution echocardiography system (Visual Sonics, Toronto, Canada). The derived echocardiography parameters included the diastolic left ventricular internal diameter (LVIDd; in mm) and systolic left ventricular internal diameter (LVIDs; in mm). The left ventricular ejection fraction (LVEF) and left ventricular fractional shortening (FS) were calculated via Visual Sonics measurement software.

### Histology and immunohistochemistry

Histology and immunohistochemistry were performed as previously described^30^. Primary antibodies against Sp1 (Millipore), PARP-1, collagen Ι and collagen III (Abcam) were used. Masson’s trichrome and Picrosirius red staining was used to detect interstitial fibrosis in the border zones. The collagen volume fraction of the border zone was analyzed by automated image analysis (Image-Pro Plus, Media Cybernetics, US).

### Terminal deoxynucleotidyl transferase-mediated dUTP nick end labeling (TUNEL) staining

Apoptosis in mice and H9c2 cells were detected by using Situ Cell Death Detection Kit (Millipore) according to the manufacturer’s instructions. At least five fields positive for apoptotic cells were counted in a blinded selection.

### Cell culture and treatments

The H9c2 cell line, purchased from the American Type Culture Collection (ATCC, Rockville, MD, USA), was cultured as previously described^11^. miR-7a/b gain or loss of expression was achieved by infecting the cells with miR-7a/b mimics and inhibitors respectively (GenePharma, Shanghai, China), using the X-treme GENE siRNA Transfection Reagent (Roche) according to the manufacturer’s instructions. The cells were treated with or without PARP-1 inhibitor 3-AB (3 mM) (Sigma) or the Sp1 inhibitor mithramycin (Cayman Chemical) before being placed in the Whitley H35 Hypoxystation (Don Whitley Scientific) for hypoxia (1% O_2_, 5% CO_2_) treatment.

### Western blotting

Western blot analysis was performed as previously described^30^. Primary antibodies against Sp1 (Millipore), PARP-1, collagen Ι and collagen III (Abcam), and caspase-3, Bcl-2, Bax (Cell Signaling Technology) were used.

### Quantitative RT-PCR

MiRs were isolated by use of TaqMan Small RNA Assay (Applied Biosystems), and cDNA synthesis involved the TaqMan MicroRNA Reverse Transcription Kit (Applied Biosystems) with RT-U6 and miR-specific stem-loop primers as described^11^. MiR-7a/b levels were measured by use of the TaqMan Universal PCR Master Mix (Applied Biosystems) in a 10-μl system. For Sp1 mRNA quantification, total RNA was homogenized in Trizol reagent (Invitrogen, CA). Complementary DNA (cDNA) was obtained by reverse transcription (Fermantas). Quantitative PCR reaction involved use of an IQ5 Real-Time PCR cycler (Bio-Rad) with the SYBR Green Reaction Kit (Bio-rad).

### Luciferase Assay

Constructs (GenePharma, Shanghai) containing the predicted binding sequence of miR-7a/b to the Sp1 3′-UTR (WT, wild type) and similar sequences with one mutated base (MU, mutant type) were separately co-transfected into H9c2 cells together with miR-7a/b mimics and scrambled siRNA. Luciferase and beta-galactosidase activity was measured using the Dual-Luciferase reporter assay system (Promega).

### Chromatin immunoprecipitation (ChIP) assay

Nucleoprotein complexes were prepared from H9c2 cells grown under either normoxia or hypoxia and treated with or without miR-7a/b mimics. ChIP was performed using EZ-ChIP^TM^ (Millipore) according to the manufacturer’s instructions. An anti-Sp1 antibody (Millipore) was used. The primers used for PCR were as follows:

PARP-1: forward, CCAGAGGCAATGAGACCAGC and reverse, ATTGCTGATGCCGGCGG; Caspase-3: forward, GGTATTGAGACAGACAGTGG and reverse, CATGGGATCTGTTTCTTTGC.

### Statistical analysis

All experiments were independently repeated at least 3 times. Data are presented as the mean ± SD and were subjected to Student’s *t* test or, where applicable, ANOVA for group comparisons using SPSS 18.0 and GraphPad Prism 6. *P* < 0.05 was considered statistically significant.

## Results

### miR-7a/b overexpression improved cardiac function

**Figure 1:**
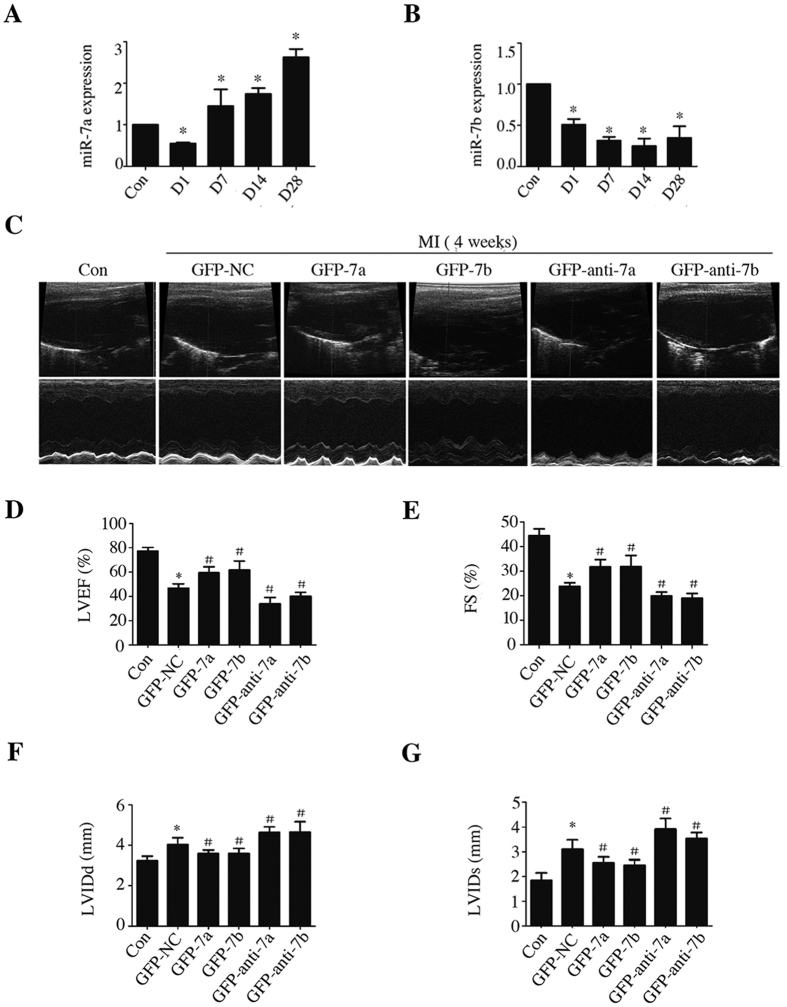
Expression levels of miR-7a/b fluctuated post MI and miR-7a/b overexpression improved cardiac function. (**A,B**) RT-PCR results represents the expression levels of miR-7a/b in the border zone of the hearts at different time after MI. (**C**) Representative 2D echocardiograms and M-mode echocardiograms 4 weeks after MI. (**D**) Left ventricular ejection fraction (LVEF). (**E**) Fractional shortening (FS). (**F**) Diastolic left ventricular internal diameter LVIDd. (**G**) Systolic left ventricular internal diameter (LVIDs). Con: sham mice without LAD occlusion. MI: mice with LAD occlusion. D: Days after MI. Data are the mean ± SD, n = 3–5/group (**A**,**B**), n = 6–7/group (**C–G**), *p < 0.05 compared with Con, ^#^p < 0.05 compared with GFP-NC.

miR-7a/b expression was quantified in the border zone of the myocardium post MI and compared to that in sham-operated mice. The decrease in miR-7a expression observed at day 1, but was less pronounced and recovered by day 7 and maintained at a high level for 4 weeks (Fig. 1A), whereas miR-7b was continuously expressed at a low level post MI (Fig. 1B).

After 4 weeks the heart was excised and immediately frozen to determine the transfection efficiency under fluorescence microscope and the PCR results further confirmed the efficiency (Fig. S1). Echocardiography showed that compared with the sham control group, the GFP-NC group exhibited cardiac dysfunction with decreased LVEF and FS values, and with expanded ventricular diameters and damaged contractile capabilities (Fig. 1C–G). miR-7a/b overexpression both elevated the LVEF and FS and diminished the LVIDd and LVIDs (Fig. 1C–G). On the contrary, compared with the GFP-NC group, the GFP-anti-7a/b groups showed further weakened cardiac function with LVEF and FS deterioration and enhanced ventricular diameter dilatation (Fig. 1C–G).

### miR-7a/b overexpression ameliorated fibrosis *in vivo*

**Figure 2:**
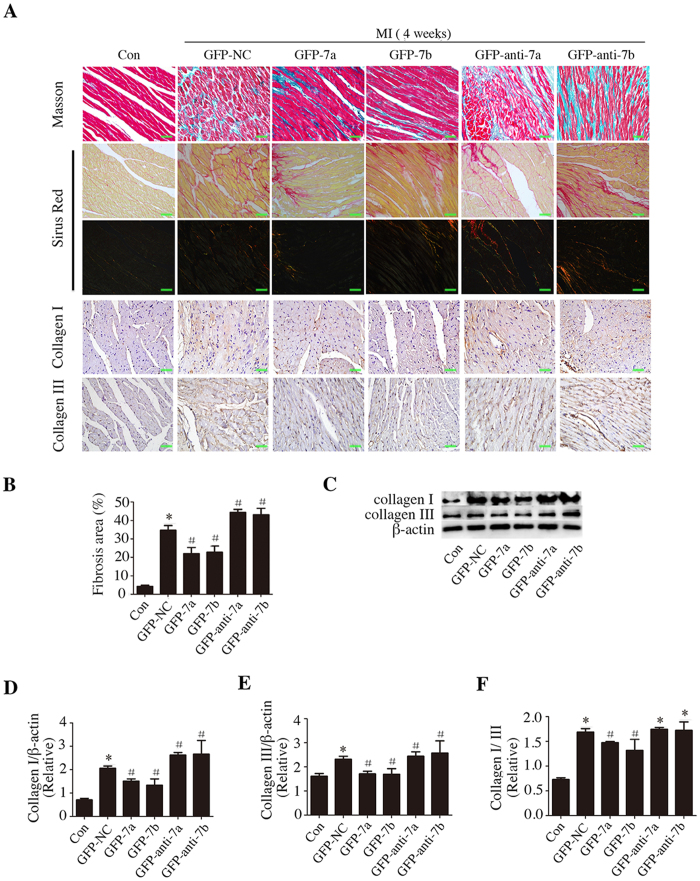
miR-7a/b overexpression ameliorated myocardial fibrosis. (**A**) Representative Masson’s trichrome staining (first row), Picrosirius red staining (second and third row), immunostaining of collagen I (fourth row) and collagen III (fifth row) (scale bar: 20 μm). (**B**) Quantitative analysis of myocardial fibrosis. (**C–F**): Western blots analysis of protein expression of collagen I and collagen III. Con: sham mice without LAD occlusion. MI: mice with LAD occlusion. (**D**) Days after MI. Data are the mean ± SD, n = 6–7/group (**A,B**), n = 3–5/group (**C–F**), *p < 0.05 compared with Con, ^#^p < 0.05 compared with GFP-NC.

We next evaluated whether the improved cardiac function was somehow due to alleviated fibrosis. Data obtained by Masson’s trichrome staining of heart sections revealed ECM (extracellular matrix) deposition in the interstitial regions in the border zone of the hearts post MI. Forced miR-7a/b expression diminished the area of collagen deposition (Fig. 2A,B). In parallel, enhanced expression of the fibrotic markers collagen I and collagen III and the increased collagen I/collagen III ratio provoked by MI were ameliorated in the GFP-7a/b groups. Whereas, knocking down miR-7a/b extended the fibrotic area and up-regulated collagen Ι expression and the collagen I/collagen III ratio (Fig. 2A,C–F).

### miR-7a/b overexpression reduced apoptosis *in vivo*

**Figure 3:**
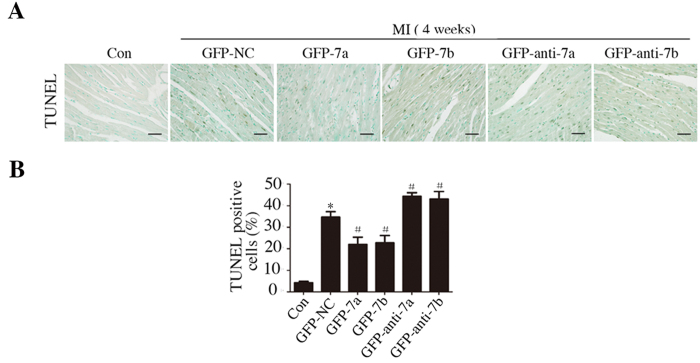
miR-7a/b overexpression reduced apoptosis of the heart. (**A**) Representative apoptosis cells in the border zones of the hearts post MI (scale bar: 20 μm). (**B**) Analysis of apoptosis cells. Con: sham mice without LAD occlusion. MI: mice with LAD occlusion. (**D**) Days after MI. Data are the mean ± SD, n = 6–7/group, *p < 0.05 compared with Con, ^#^p < 0.05 compared with GFP-NC.

Compared with the control group, MI induced apoptosis in the border zone and the restoration of miR-7a/b reduced TUNEL-positive cells, whereas the GFP-anti-7a/b groups processed more apoptotic cells (Fig. 3A,B).

### miR-7a/b repressed PARP-1 and *in vivo* and *in vitro*

**Figure 4:**
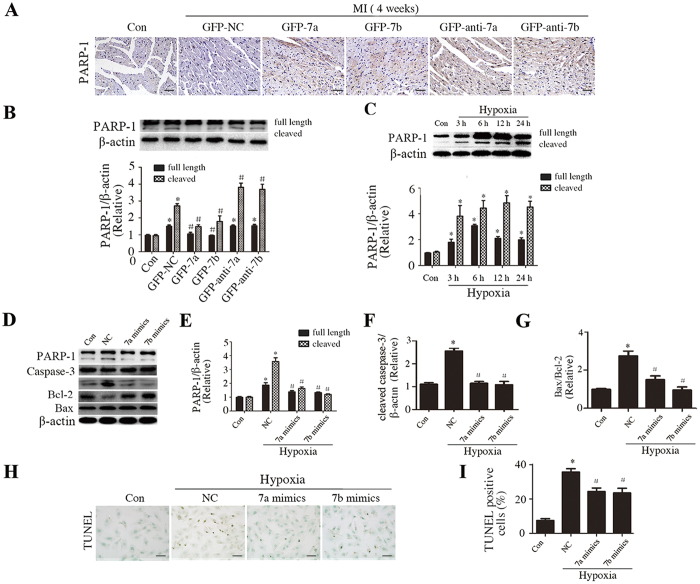
miR-7a/b repressed of PARP-1 expression *in vivo* and *in vitro*, and reduced apoptosis *in vitro*. (**A**) Immunostaining of PARP-1 *in vivo*. (**B**) Western blots of PARP-1 *in vivo*. (**C**) Western blots of PARP-1 in cells exposed to hypoxia for different time. (**D–G**) Western blots showing the effect of miR-7a/b on regulation of PARP-1 (**D**,**E**), cleaved caspase-3 (**D,F**), Bax/Bcl-2 (**D**,**G**). (**H,I**) TUNEL assay results (Scale bar: 50 μm). Con: sham mice without LAD occlusion (**A,B**) or normal cultured H9c2 cells (**C–H**). MI: mice with LAD occlusion. NC: H9c2 cells exposed to hypoxia for 12 h. Data are the mean ± SD, n = 6/group (**A**), n = 3/group (**B–I**), *p < 0.05 compared with Con; ^#^p < 0.05 compared with GFP-NC (**A,B**) or NC (**C–I**).

**Figure 5:**
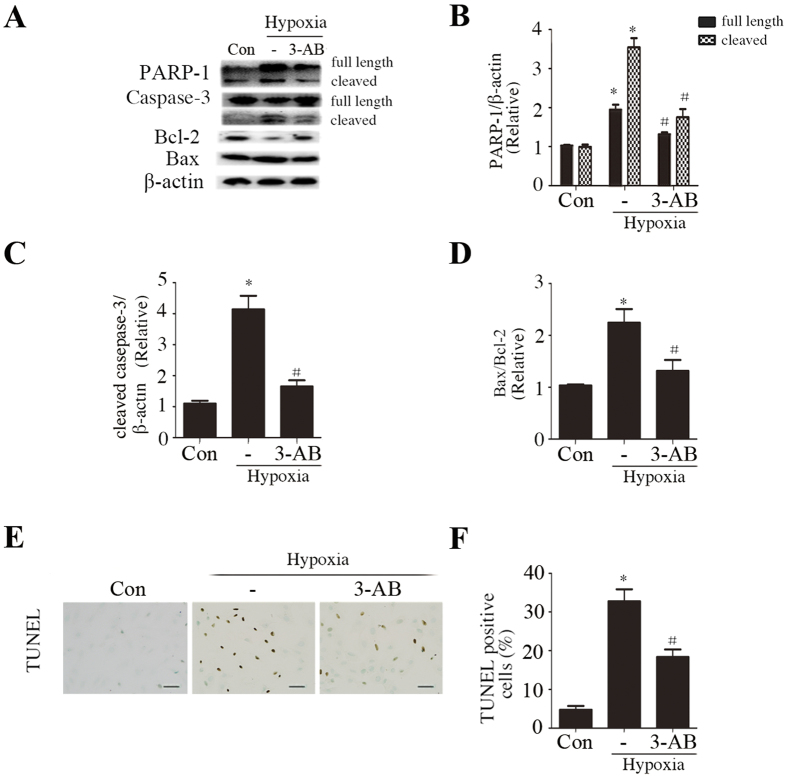
3-AB decresed PARP-1 expression and apoptosis *in vitro*. (**A–D**) Western blots showing the effect of 3-AB on regulation of PARP-1 (**A,B**), cleaved caspase-3 (**A,C**), Bax/Bcl-2 (**A,D**). (**E,F**) TUNEL assay results (Scale bar: 50 μm). Con: normal cultured H9c2 cells. –, cells only exposed to hypoxia. Data are the mean ± SD, n = 3/group, *p < 0.05 compared with Con; ^#^p < 0.05 compared with-.

To determine the possible underlying cellular mechanisms for the protective effects of miR-7a/b, we further studied the expression of PARP-1, a well-confirmed target, in the border zone of MI heart sections. Overexpressed of miR-7a/b effectively repressed the MI-stimulated PARP-1 expression while silence of miR-7a/b even further stimulated PARP-1 (Fig. 4A,B). We also assessed the expression of PARP-1 and the effect of miR-7a/b in hypoxic H9c2 cells. During hypoxia, PARP-1 expression increased in a time-dependent manner and the maximum effect was observed at 12 hours (Fig. 4C), which also effectively stimulated caspase-3 and Bax expression while inhibited bcl-2 expression (Fig. 4D). Therefore, we chose 12 h for the following experiment. As miR-7a/b expression was up-regulated and no significant difference of miR-7b after exposure to hypoxia (Fig. S2A), we induced miR-7a/b overexpression/silence by transfection with miR-7a/b mimics/inhibitors into H9c2 cell and the transfection efficiencies are presented in Fig. S2B,C. As predicted, miR-7a/b mimics significantly alleviated the repressed expression of PARP-1, cleaved caspase-3, and the ratio of Bax/Bcl-2 (Fig. 4D–G), as well as hypoxia-induced apoptosis as confirmed by TUNEL assays (Fig. 4H,I),

Further, pretreated with 3-AB profoundly decreased the expression of PARP-1, cleaved caspase-3 and the ratio of Bax/Bcl-2 (Fig. 5A–D), and saved cells from hypoxia-induced apoptosis (Fig. 5E,F).

### miR-7a/b repressed Sp1 expression and directly targeted Sp1

**Figure 6:**
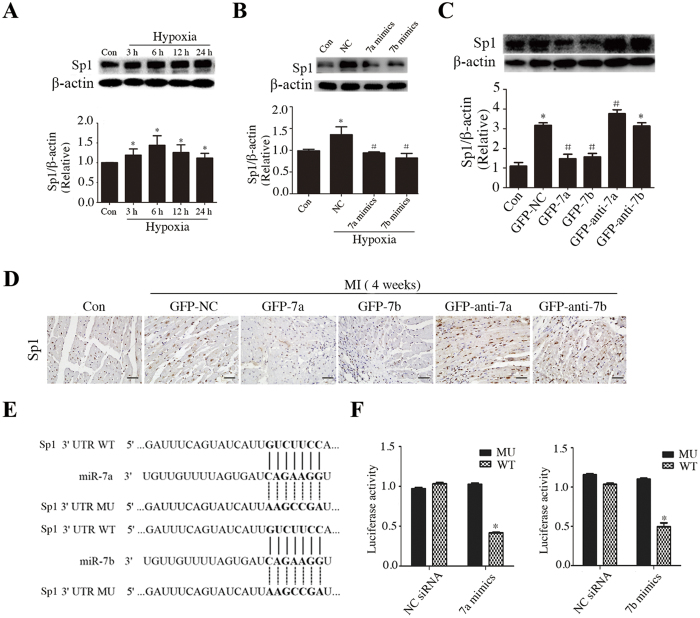
miR-7a/b repressed the downstream target Sp1 expression *in vivo* and *in vitro*. (**A**) Western blots of Sp1 in cells exposed to hypoxia for different time, (**B,C**) Western blots showing the effect of miR-7a/b on regulation of Sp1 *in vitro* (**B**) and *in vivo* (**C**). (**D**) Immunostaining of Sp1 *in vivo* (scale bar: 20 μm). (**E**) Conserved miR-7a/b binding sites and mutated binding sites in 3′ untranslated region (UTR) of Sp1. (**F**) Luciferase activity analysis. Con: sham mice without LAD occlusion. MI: mice with LAD occlusion. Con: normal cultured H9c2 cells (**A,B**) or sham mice without LAD occlusion (**C,D**). MI: mice with LAD occlusion. NC: H9c2 cells exposed to hypoxia for 12 h. Data are the mean ± SD, n = 3/group (**A–C**,**F**), n = 6/group (**D**), *p < 0.05 compared with Con (**A–C**) or NC siRNA. (F); ^#^p < 0.05 compared with NC (**A,B**) or GFP-NC (**C,D**).

Hypoxia also activated Sp1 expression, which is down-regulated by miR-7a/b in H9c2 cells Fig. 6A,B. Similarly, GFP-7a/b groups presented decreased Sp1 expression when compared to MI group, whereas the GFP-anti-7a group showed increased expression, and no significant differences were observed between the GFP-NC and GFP-anti-7b groups (Fig. 6C,D). Moreover, miR-7a mimics repressed luciferase activity and miR-7b mimics performed similarly (Fig. 6E,F), providing evidence of the direct binding of miR-7a/b to the Sp1 3′-UTR.

### Sp1 binding activity was crucially involved in miR-7a/b-regulated PARP-1 and caspase-3 expression *in vitro*

**Figure 7:**
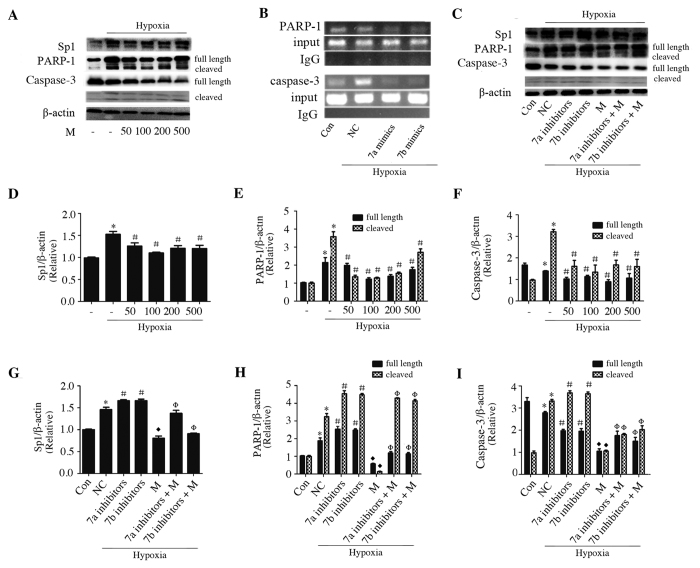
Sp1 binding activity mediated miR-7a/b-regulated Sp1, PARP-1 and caspase-3 expression in hypoxia H9c2 cells. (**A,D–F**) Western blots of Sp1, PARP-1 and caspase-3 in hypoxic cells pretreated with different concentration of mithramycin (nM). (**C,G–I**) Western blots of Sp1, PARP-1 and caspase-3 in hypoxic H9c2 cells transfected with miR-7a/b inhibitors that treated with or without 100 nM mithramycin. (**B**) Representative ChIP assays. Con: normal cultured H9c2 cells, M: mithramycin, *p < 0.05 compared with control group, ^#^p < 0.05 compared with NC siRNA-transfected group, ^♦^p < 0.05 compared with miR-7a/b inhibitors-transfected group; ^Φ^p < 0.05 compared with mithramycin-treated group.

We used mithramycin, the inhibitor of Sp1, to clarify the Sp1 binding activity that maybe involved in hypoxic cells. Mithramycin, negatively regulated Sp1, PARP-1 and caspase-3 in a concentration-dependent manner (Fig. 7A,D–F), which suggests a crucial role of Sp1 binding activity in mediating miR-7a/b function. The maximum effect were observed at 100 nM, therefore, this concentration was used for subsequent experiments. Compared to NC group, pre-transfected with miR-7a/b inhibitors effectively up-regulated Sp1, PARP-1 and caspase-3 expression, however, pretreatment of mithramycin before hypoxia, meaningfully counteracted the effects caused by miR-7a/b inhibitors (Fig. 7C,G–I), suggesting that miR-7a/b directly modulates Sp1 and that the binding activity of Sp1 may conditionally mediate the repression of miR-7a/b-regulated PARP-1 and caspase-3 expression.

ChIP assays were conducted to confirm that the binding activity of Sp1 was involved in the regulation of PARP-1 and caspase-3. As shown in the representative ChIP blot results in Fig. 7B, miR-7a/b disturbed hypoxia-increased Sp1 DNA binding activity of both PARP-1 and caspase-3 promoters, suggesting that Sp1 mediates miR-7a/b-regulated PARP-1 and caspase-3 expression in hypoxic H9c2 cells.

## Discussion

In the current study, we overexpressed miR-7a/b in the heart to identify a potential strategy to ameliorate post-MI cardiac dysfunction. As expected, miR-7a/b overexpression improved cardiac function, decreased the fibrosis *in vivo*, and reduced apoptosis both *in vivo* and *in vitro*. Additionally, miR-7a/b directly targeted Sp1, which then appeared to mediate miR-7a/b-regulated PARP-1 and caspase-3 expression. These results demonstrate the cardio-protective role of miR-7a/b in response to MI and hypoxia.

In the early phase of acute MI, miRs deregulation is likely induced by irretrievable cell death and severe oxidative stress, whereas in later stages, miR regulation is likely associated with cardiac remodeling and functions as a compensatory mechanism. Endogenously expressed miR-7a was initially down-regulated in the myocardium following MI and was subsequently restored, whereas miR-7b was maintained at low levels. Therefore, miR-7a/b may participate in post-infarction remodeling. We overexpressed miR-7a/b and found ameliorated cardiac function, relieved apoptotic injury and narrowed fibrotic area post MI, which was consistent with our previous study reporting that miR-7a/b profoundly minimized the infarction size during I/R injury and protected cardiomyocytes from apoptosis^11^. Mechanistically, we previously found miR-7a/b exhibited an anti-fibrotic effect in angiotensin II-stimulated cardiac fibroblast by targeting collagen I in Sp1-dependent manner^31^. As post-infarction cardiac dysfunction is determined by the extent of remodeling, particularly apoptosis and fibrosis^4^,^32^, the effects of miR-7a/b on apoptosis and fibrosis could contribute to the improved cardiac function post MI.

As an abundant nuclear enzyme and an identified target of miR-7a/b, PARP-1 plays a pivotal role in DNA repair and apoptosis under cardiac stress^15^, and the 116-kDa PARP-1 protein is cleaved into an 89-kDa apoptotic fragment during apoptosis^33^. Others and we found that PARP-1 over-activation harmed the myocardium, and the genetic deletion of PARP-1 or pharmacological inhibition of its activity reduced the infarcted area and apoptosis, as well as prevented the expression of fibrotic and inflammatory genes^11^,^13^,^14^,^15^,^16^,^17^. In current experiment, miR-7a/b overexpression profoundly down-regulated PARP-1 both *in vivo* and *in vitro*. Accordingly, the repression of PARP-1 expression could serve as another explanation of the protective role of miR-7a/b in regulating post-MI remodeling.

To find other possible targets of miR-7a/b involved in MI, we used TargetScan and predicted miR-7a/b binding sites at the Sp1 3′UTR, and transfection with miR-7a/b mimics significantly inhibited Sp1 expression and resulted in a decreased luciferase activity of GV126-Sp1-3′UTR-WT. Therefore, Sp1 is another functional target gene of miR-7a/b in H9c2 cells.

In the current study, the inhibition of Sp1 DNA binding activity by treatment with mithramycin remarkably reduced pro-apoptotic PARP-1 and caspase-3 expression, suggesting Sp1 binding activity maybe involved in the miR-7a/b-regulated PARP-1 and caspase-3 exprssion. As Sp1 could regulate PARP-1^19^,^27^ and caspase-3 promoter activity^20^, we further performed ChIP and revealed an increased association between Sp1 and the PARP-1 promoter during hypoxia to clarify our hypothesis. Similar to the expression, the binding capability of Sp1 to caspase-3 promoter was also stimulated by hypoxia and abrogated by miR-7a/b. Therefore, Sp1 may be a functional modulator of PARP-1 and caspase-3 expression in hypoxic cardiac myocytes, and the effects of miR-7a/b in the regulation of PARP-1 and caspase-3 appeared to be partially mediated by its novel target, Sp1. In addition to PARP-1 and caspase-3, Sp1 regulates other apoptotic molecules as well as fibrotic and inflammatory molecules. For example, Sp1 is involved in the excessive expression and subsequent deposition of collagen I 24,34,35 as well as in the regulation of TNF-α^26^, NF-κB and Cox-2^25^. Pharmacological therapy to postpone cardiac remodeling after MI always inhibits the activity of Sp1. Angiotensin II receptor blocker (ARB) and angiotensin-converting enzyme inhibitor (ACEI) remarkably prevent Sp1 DNA binding activity in the infarcted area post MI^36^, suggesting that miR-7a/b may act similarly to these drugs and may be potentially suitable as an intervention in MI.

It is interesting to notice that miR-7a/b stimulated Bcl-2 expression in hypoxic H9c2 cells, whereas Bcl-2 was found to be a target of miR-7^37^. One possible explanation to this maybe that the affinity of miR-7 with its target is not identical in different cells under various circumstances and during hypoxia, Bcl-2 is largely repressed and miR-7a/b preferentially bind to Sp1 and Parp-1. Another explanation may contribute to the integrated role of miR-7a/b by targeting their multiple targets, such as IGF-1, IRS-1, EGFR, c-fos, Cox-2 and so on. With the unsolved problems associated with accurately identifying the precise role of Sp1 in mediating the function of miR-7a/b *in vivo*, further investigations are necessary. Additionally, whether the MI-induced deregulation of miR-7a/b has other, more beneficial consequences via the regulation of currently unknown targets remains to be determined.

## Conclusions

In conclusion, miR-7a/b collectively exerted anti-apoptotic effects and saved failing hearts after MI. As the clinical outcome of MI is determined by numerous mediators and signaling pathways, the appropriate and timely modulation of miR expression may be useful for the development of an entire potent class of drugs for heart disorders.

## Additional Information

**How to cite this article**: Li, R. *et al*. miR-7a/b attenuates post-myocardial infarction remodeling and protects H9c2 cardiomyoblast against hypoxia-induced apoptosis involving Sp1 and PARP-1. *Sci. Rep*. **6**, 29082; doi: 10.1038/srep29082 (2016).

## Supplementary Material

Supplementary Information
