# Supplementary material for: miR-7a/b attenuates post-myocardial infarction remodeling and protects H9c2 cardiomyoblast against hypoxia-induced apoptosis involving Sp1 and PARP-1
Source: Sci Rep. 2016 Jul 7;6:29082. doi: 10.1038/srep29082 (PMC4935883; doi:10.1038/srep29082)
Supplement: Supplementary Information [file srep29082-s1.pdf]

miR-7a/b attenuates post-myocardial infarction remodeling and protects H9c2 cardiomyoblast against hypoxia-induced apoptosis involving Sp1 and PARP-1

Rui Li[a,b,1], Hai-hua Geng[a,1], Jie Xiao[a,], Xiao-teng Qing[a], Fu Wang[a], Jun-hui Xing[a], Yan-fei Xia[a], Yang Mao[a], Jing-wen Liang[a], Xiao-ping Ji[a,]*

[a] The Key Laboratory of Cardiovascular Remodeling and Function Research, Chinese Ministry of Education and Chinese Ministry of Public Health, Qilu Hospital of Shandong University, Jinan, Shandong, China

[b] Department of Health Care, China-Japan Friendship Hospital, Ministry of Health, Beijing, China

[1] These authors contributed equally to this work.

* Corresponding author. Tel.: +86 531 82169429; fax: +86 531 86169356. E-mail address: jxp1964@hotmail.com

Supplementary information list

1. Supplementary figure 1 and figure legend.

2. Supplementary figure 2 and figure legend.

1. Supplementary figure 1

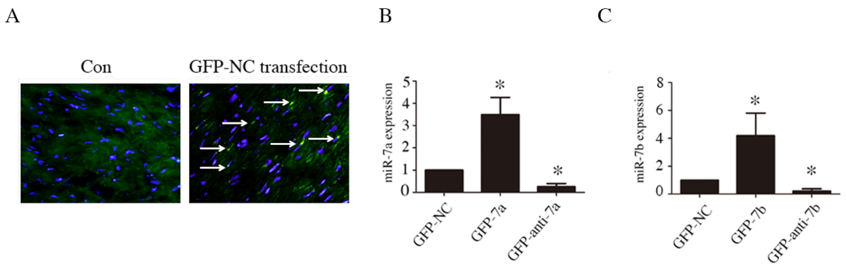

Fig. S1. (A) **Representative fluorescence microscopy of GFP-labeled scramble transfection efficiency in mouse myocardial tissue** (original magnification ×400). (B, C) The efficacy of miR-7a/b mimics/inhibitors was determined by qRT-PCR. *$p < 0.05$ compared with control.

2. Supplementary figure 2

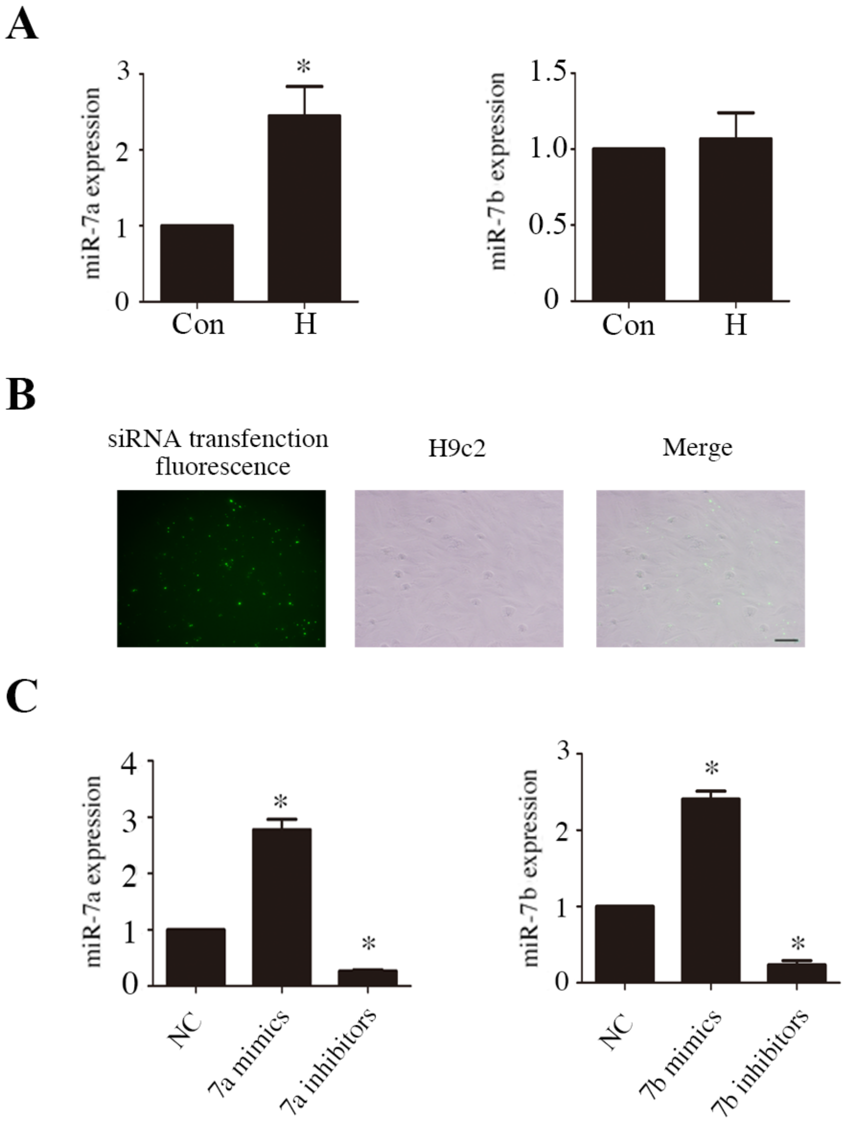

Fig. S2. Expression of miR-7a/b in H9c2 cells and transfection efficiency in into H9c2 cells. (A) Expression of miR-7a/b in cells that under hypoxia for 12 h. (B) The transfection efficacy of siRNA into H9c2 cells (Scale bar: 50 μm). (C) The efficacy of miR-7a/b mimics/inhibitors was determined by qRT-PCR. *$p < 0.05$ compared with control.
